# Isolated breast hydatid cyst: Imaging features

**DOI:** 10.1002/ccr3.6362

**Published:** 2022-09-20

**Authors:** Mazhoud Ines, Ben Lassoued Mariem, Moussaoui Marwa, Ben Salem Amina, Hafsa Chiraz

**Affiliations:** ^1^ Radiology department of the maternity and neonatalology center in Moanstir Monastir Tunisia

**Keywords:** breast, hydatid cyst, magnetic resonance imaging, mammography, ultrasound

## Abstract

Hydatid cyst (HC) of the breast is a rare entity, even in endemic areas. We report the radiologic features of an isolated breast HC in a 50‐year‐old woman. Imaging findings may mimic other common breast lesions, but specific imaging features help establish an accurate diagnosis to adapt therapeutic management.

## Abstract 2

Although very rare, hydatid cyst of the breast should be included in differential diagnosis of breast lumps, especially for patients living in endemic areas. Imaging findings are essential to evoke this potentially serious breast disease in order to adapt therapeutic management.

## 1 INTRODUCTION

HC is a parasitosis caused by the larval form of Echinococcus granulosis. It commonly affects the liver (75%) and lungs (15%). The breast is a very rare location. It accounts for 0.27%; it may be affected as a part of disseminated disease, and may, in rare cases, be a site of primary disease.^1^

Breast HC may mimic other benign lesions or, in rare cases, malignant tumors which make the diagnosis more challenging. Ultrasound and magnetic resonance imaging (MRI) have a pivotal role in the diagnosis and management of this rare entity.^2^

## 2 CASE REPORT

A 50‐year‐old woman, originally from a rural area, with no particular medical or surgical history, presented, on the gynecology department, with a painless left breast lump. She reported a gradual increase in size over the last few years. There was no history of fever, nipple discharge, or family history of breast cancer. Physical examination revealed a firm, non‐mobile lump in the lower quadrants of the left breast, fixed to the deep tissue plan, with well‐defined margins, measuring about 7 cm. There were no local inflammatory signs or locoregional adenopathy.

The right breast was normal, and a systemic examination did not show any abnormality.

**FIGURE 1:**
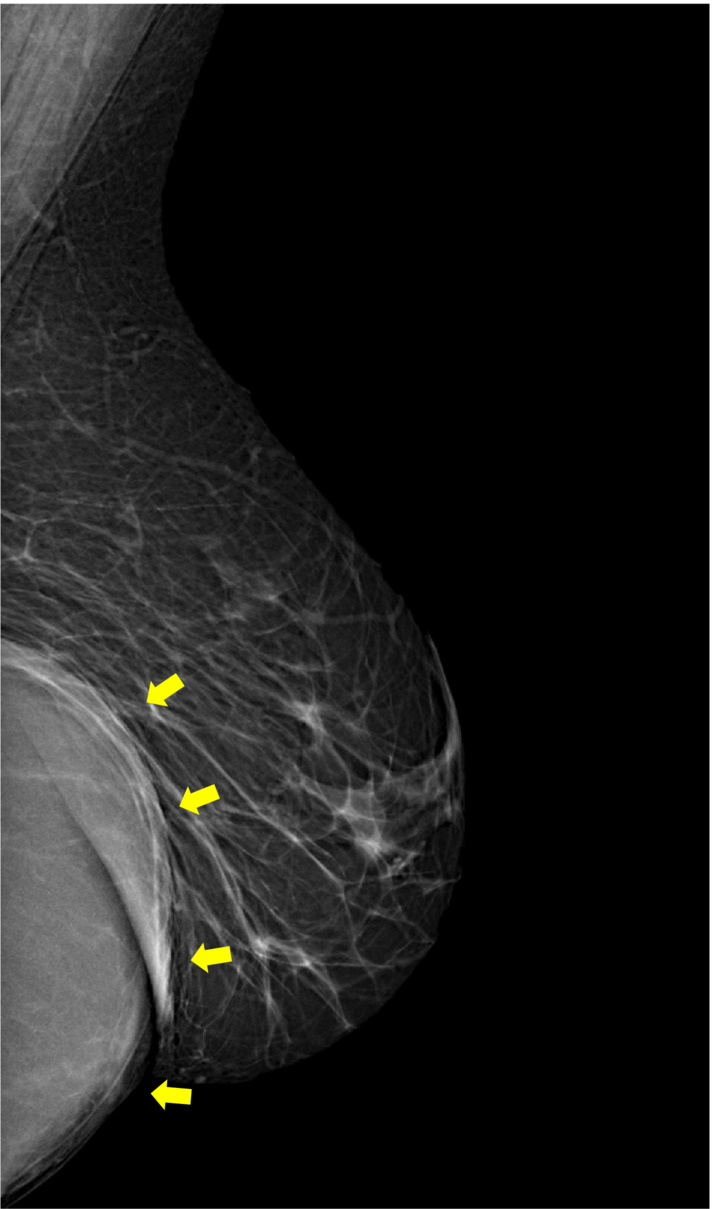
Medio‐lateral oblique view mammography (MLO) of the left breast shows a deep lesión, with well‐defined anterior contours (yellow arrow) and hidden posterior contours, in the inframammary fold and the lower quadrants (arrows); no calcification or architectural distortion is noticed

Mammography, with craniocaudal (CC) and medio‐lateral oblique (MLO) views, showed a well‐circumscribed mass in the inframammary fold and the lower quadrants of the left breast. Its density was homogeneous, and there were no identifiable micro‐ and macrocalcifications, architectural disorganization, or skin thickening (Figure 1)

**FIGURE 2:**
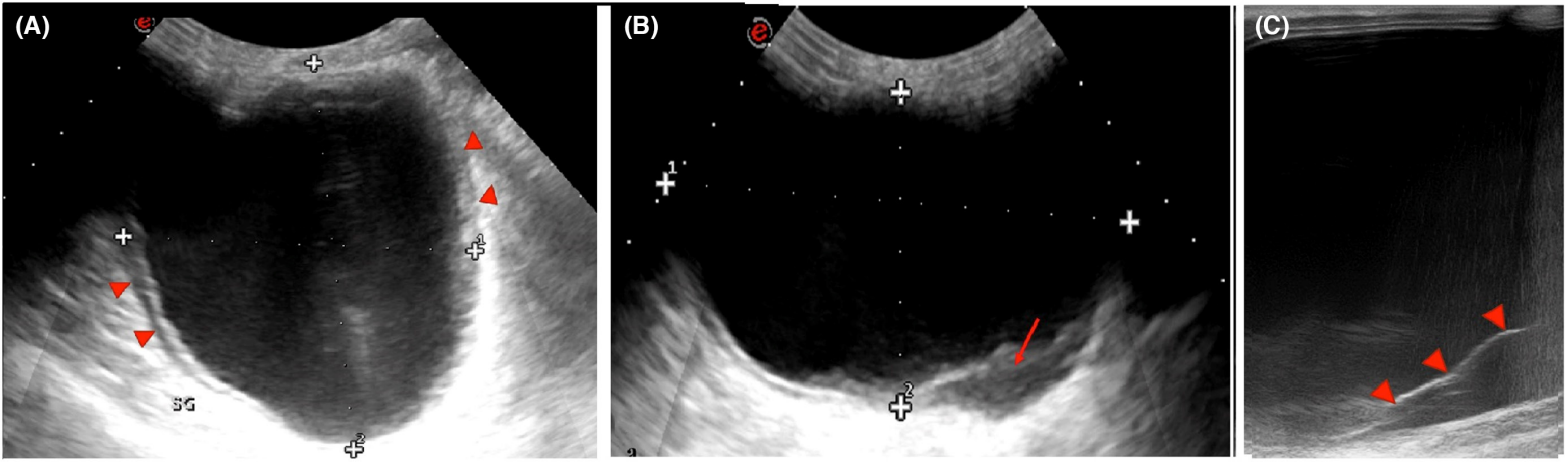
Ultrasound of the left breast shows: *(A)* a unilocular anechoic cystic lesion, with a thickened wall(arrowheads); *(B)* an echogenic sediment consistent with hydatid sand (arrow); and *(C)* a detached posterior membrane(arrowheads)

The ultrasound showed an anechoic unilocular cystic mass, thick‐walled, containing an internal detached membrane associated with echogenic sediment, without any internal vascularity on Doppler sonography. There were no significant enlarged axillary lymph nodes detected (Figure 2).

**FIGURE 3:**
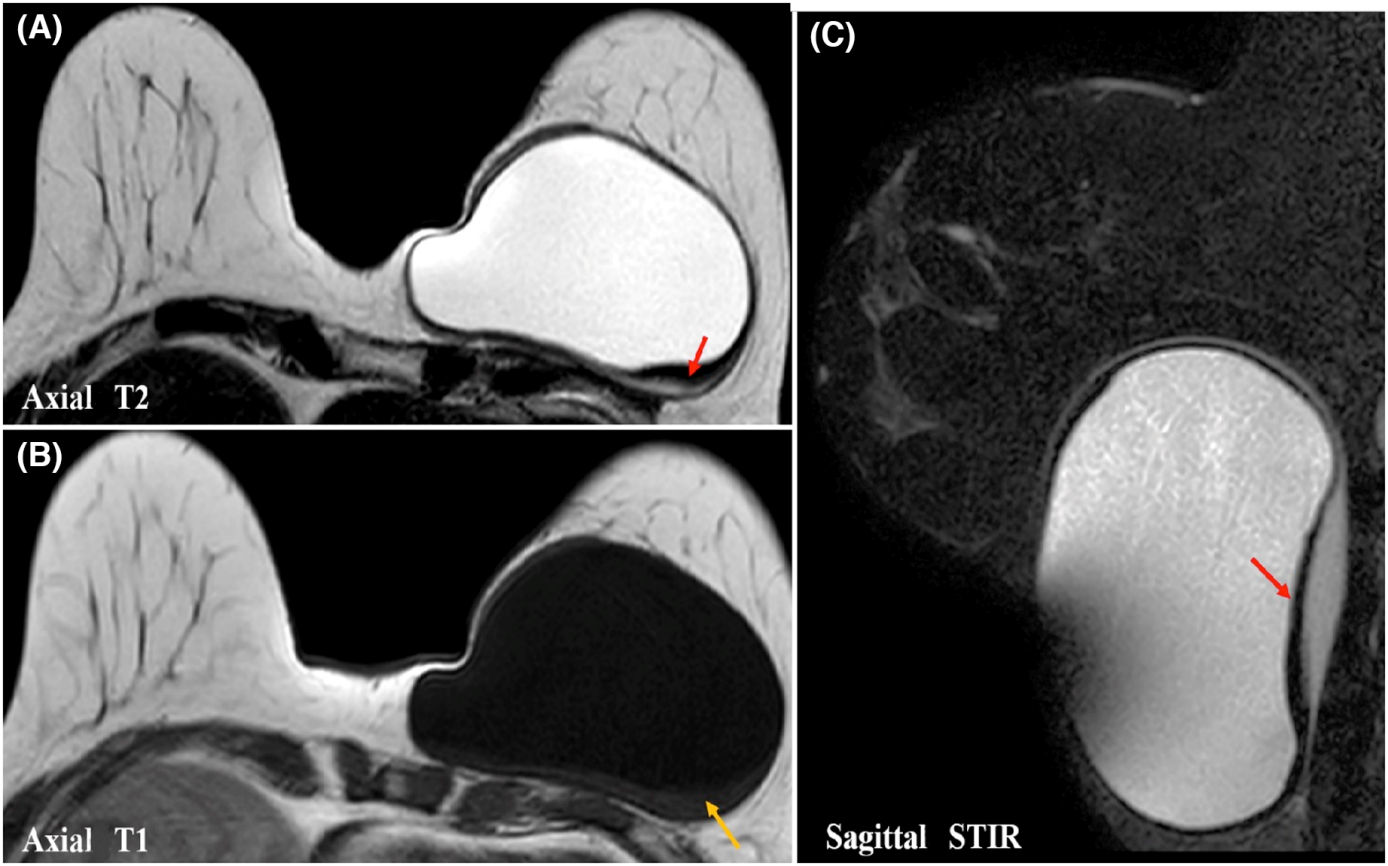
Left breast MRI: Axial T2 *(A)*, T1 *(B),* and sagittal STIR *(C)* weighted images show a cystic lesion with high signal on T2/STIR and low signal on T1. A posterior detachment of the germinal membrane which appears hypointense on T2 and STIR sequences (red arrow). The presence of a sedimentary material with intermediate T1 signal intensity is compatible with hydatid sand (yellow arrow)

**FIGURE 4:**
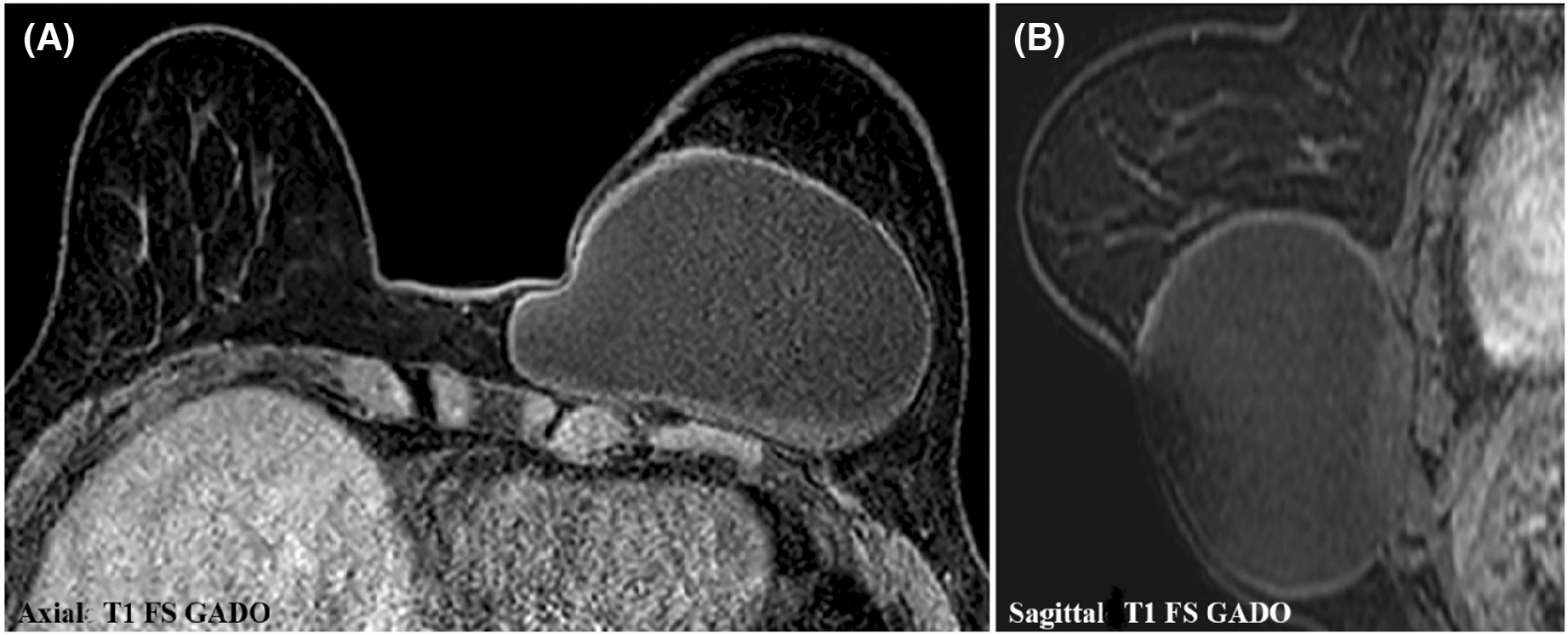
Axial *(A)* and sagittal *(B)* contrast‐enhanced T1‐weighted fat‐suppressed MR images show a regular enhancement of the capsular wall

On breast MRI, the cystic lesion appeared hyperintense on T2‐weighted and STIR (short T1 inversion recovery) MR sequences, hypointense on T1‐weighted sequences. The capsular wall was smooth, moderately thickened and had low‐signal‐intensity on T2‐ and STIR‐weighted images with regular enhancement on dynamic T1‐weighted images. The detached membrane had a low signal intensity in all sequences. We noted a T1 isointense sedimentary material without enhancement compatible with hydatid sand (Figures 3 and 4)

Those imaging findings suggested the diagnosis of HC. No other hydatid lesions were found on the abdominopelvic ultrasound and the chest X‐ray. The hydatid serology was negative.

Given the radiological results, the diagnosis of hydatid cyst was strongly suspected. Faced with the contraindication of microbiopsy in this case, we opted for surgical resection.

A surgical excision of the cyst was performed without intraoperative complications, and the post‐operative histopathological results confirmed the diagnosis of HC.

Preoperative albendazole was administered. No treatment after chirurgy was advised because no intraoperative rupture was declared. Follow‐up 6 months, no evidence of recurrence was shown.

## 3 DISCUSSION

Hydatid disease is a zoonosis caused by the larvae of Echinococcus granulosus. Although most prevalent in underdeveloped countries, hydatid cysts have a world‐wide distribution.^3^ Humans are accidental intermediate hosts. The parasite ingested enters the portal circulation. The liver acts as the first filter and stops about 75% of the embryos, followed by the lung. Only about 15% of the embryos develop cysts in other organs of the body. Hydatid cysts of the breast usually occur via hematogenous spread.^4^

There is no specific clinical presentation of breast HC. Patients usually present with a palpable and painless lump in the breast gradually increasing in size.^2^ The cyst inflammation may cause skin thickening and axillary lymphadenopathy, mimicking mastitis, and in particular, inflammatory breast cancer.^5^

On mammography, a hydatid cyst is usually shown as a homogenous, well‐circumscribed mass with possible peripheral or internal calcifications.^5^ This aspect can also suggest other benign lesions such as a cyst, fibroadenoma, phyllodes tumor, and chronic abscess.^6^ Vega et al. noted that the presence of ring‐shaped structures inside the mass should suggest a hydatid cyst. They can be explained by the difference in the density of the wall and the contents of vesicles within the main cyst.^1^

Ouedraogo et al. studied the mammographic aspects of 20 cases of hydatic cysts and proposed a radiological classification; type1: dense, rounded mass, well‐limited, with no calcifications. Type 2: dense, rounded mass, surrounded by peripheral thin calcifications. Type 3: total calcified rounded mass. Type 4: dense, rounded, and circumscribed mass, with scattered macroalcifications). The most common radiological aspects are Types 2 and 3.^7^ Our case showed mammographic features suggestive of a type 1 cyst.

The ultrasonographic findings change according to the stage of development of the parasite. The appearance of HC can vary from an anechoic unilocular cyst to a lobulated mass with heterogeneous echostructure. This appearance can mimic benign and malignant lesions, hence the interest in evoking it in endemic areas^5^ Several ultrasound signs suggestive of HC have been described. The most common is the “double‐wall” sign, where the cyst wall is seen as two echogenic layers. The complete detachment of the endocyst from the pericyst leads to a floating membrane which produces the “water lily” sign.^6^ A fluid level with moving echoes may be seen due to hydatid sand composed of hooklets, membranes, and debris, which gives the appearance of the “Snowstorm”.^8^ Another sign, known as the “congealed water lily” sign, has been described as strongly suggestive of hydatid cysts. It appears as an increased echogenicity of cyst fluid (secondary to its increased viscosity) and an immobile germinal membrane, giving it a solid appearance.^9^

Gharbi et al. have described five types of ultrasound aspects for hydatid cysts^10^:(Type 1: Unilocular simple cyst; Type2: cyst with detached membrane; Type 3: multivesicular multiseptated cyst; Type 4: heterogeneous echo patterns with pseudo‐solid appearance, and Type 5: cyst with calcified thick wall), HC types 2 and 3 have more reliable diagnostic imaging properties than other types. In our case, we noted the presence of both membrane detachment and an echogenic sediment suggestive of a type 2 cyst.

The use of breast MRI in the diagnosis of hydatid cyst allows a more detailed analysis of the internal structure of the HC. A T2 low‐signal‐intensity rim “rim sign” has been described as characteristic of HC. This corresponds to the parasitic membranes and pericyst, which is a fibrous capsule.^6^ Internal floating membranes also have low signal intensity in all sequences, which is another specific imaging feature of a HC.^11^ Hydatid sand has an intermediate T1 signal intensity. Daughter vesicles may appear hypointense or isointense relative to the maternal matrix on T1‐ and T2‐weighted MR images. Capsular enhancement can be seen, even in the absence of infection, and it may be related to sterile inflammation. It may be difficult to distinguish a HC from a breast abscess or an inflammatory carcinoma, hence the importance of clinical examination^5^, ^6^ In our case, the MRI showed a membrane detachment, as well as a sediment of the hydatic sand isointense on T1‐weighted images.

The enzyme‐linked immunosorbent assay (ELISA) test may also be used to confirm the hydatid nature of the lesion. It is highly sensitive and specific for liver HC but less sensitive and specific in other locations.^12^

The preoperative diagnosis of breast HC is essential for taking the necessary precautions to minimize the risk of peroperative rupture. A total excision of the cyst without any spillage of hydatid material is the only curative treatment.^13^

## 4 CONCLUSION

Breast HC is uncommon. However, it should be included in the differential diagnosis of breast lump, especially in endemic areas. Our case shows characteristic radiologic findings that help to diagnose this rare entity and distinguish it from other breast lesions, thus allowing appropriate therapeutic management.

## AUTHOR CONTRIBUTIONS

Mazhoud Ines involved in collecting the data, organization. Ben Lassoued Meriem involved in writing–original draft. Moussaoui Marwa involved in validation. Ben Salem Amina contributed to resources. Chiraz Hafsa involved in investigation.

## CONFLICTS OF INTEREST

The authors have no conflicts of interest to declare.

## CONSENT

Written informed consent was obtained from the patient to publish this report in accordance with the journal's patient consent policy. The data that support the findings of this study are available in this article.
